# CD4 cell count recovery among HIV-infected patients with very advanced immunodeficiency commencing antiretroviral treatment in sub-Saharan Africa

**DOI:** 10.1186/1471-2334-6-59

**Published:** 2006-03-21

**Authors:** Stephen D Lawn, Landon Myer, Linda-Gail Bekker, Robin Wood

**Affiliations:** 1 The Desmond Tutu HIV Centre, Institute for Infectious Disease and Molecular Medicine, Faculty of Health Sciences, University of Cape Town, Anzio Road, Observatory 7925, Cape Town, South Africa; 2 Clinical Research Unit, Department of Infectious and Tropical Diseases, London School of Hygiene and Tropical Medicine, Keppel Stree, London, UK; 3 Infectious Diseases Epidemiology Unit, School of Public Health and Family Medicine, Faculty of Health Sciences, University of Cape Town, Anzio Road, Observatory 7925, Cape Town, South Africa; 4 Department of Epidemiology, Mailman School of Public Health, Columbia University, New York, USA

## Abstract

**Background:**

Patients accessing antiretroviral treatment (ART) programmes in sub-Saharan Africa frequently have very advanced immunodeficiency. Previous data suggest that such patients may have diminished capacity for CD4 cell count recovery.

**Methods:**

Rates of CD4 cell increase were determined over 48 weeks among ART-naïve individuals (n = 596) commencing ART in a South African community-based ART programme.

**Results:**

The CD4 cell count increased from a median of 97 cells/μl at baseline to 261 cells/μl at 48 weeks and the proportion of patients with a CD4 cell count <100 cells/μl decreased from 51% at baseline to just 4% at 48 weeks. A rapid first phase of recovery (0–16 weeks, median rate = 25.5 cells/μl/month) was followed by a slower second phase (16–48 weeks, median rate = 7.7 cells/μl/month). Compared to patients with higher baseline counts, multivariate analysis showed that those with baseline CD4 counts <50 cells/μl had similar rates of phase 1 CD4 cell recovery (P = 0.42), greater rates of phase 2 recovery (P = 0.007) and a lower risk of immunological non-response (P = 0.016). Among those that achieved a CD4 cell count >500 cells/μl at 48 weeks, 19% had baseline CD4 cell counts <50 cells/μl. However, the proportion of these patients that attained a CD4 count 200 cells/μl at 48 weeks was lower than those with higher baseline CD4 cell counts.

**Conclusion:**

Patients in this cohort with baseline CD4 cell counts <50 cells/μl have equivalent or greater capacity for immunological recovery during 48 weeks of ART compared to those with higher baseline CD4 cell counts. However, their CD4 counts remain <200 cells/μl for a longer period, potentially increasing their risk of morbidity and mortality in the first year of ART.

## Background

The World Health Organisation (WHO) estimated that in June 2005 4.7 million people living in sub-Saharan Africa were in urgent need of antiretroviral treatment (ART) [1]. Despite formidable logistical hurdles, the number of individuals able to access this treatment in the region is expanding. One of the programmatic challenges facing ART services in sub-Saharan Africa is that many HIV-infected patients only access healthcare once they have developed advanced symptomatic disease [2] and this delay may be further compounded by health-systems delays. The median CD4 cell count among those enrolling in ART programmes is often under 100 cells/μl even where programmes have been well established for several years [3-5].

Patients enrolling into ART programmes with very low CD4 cell counts have heightened risk of morbidity and mortality both before and during the initial months of ART [5-8]. Moreover, advanced pre-treatment immunodeficiency is also reported to be associated with diminished capacity for restoration of CD4 cell counts and CD4 cell functional responses during ART [9-14]. This raises the concern that many patients entering ART programmes in sub-Saharan Africa may have limited potential for immune recovery. Although previous studies from sub-Saharan Africa have reported overall CD4 cell count responses [3,15,16], there are no published data from the region regarding rates of CD4 cell recovery and rates of immunological non-response to ART among patients with CD4 cell <50 cells/μl.

In this study we have examined determinants of CD4 cell count recovery among patients accessing a community-based antiretroviral programme in South Africa. We focus on the hypothesis that advanced pre-treatment immunodeficiency diminishes the capacity for CD4 cell count recovery during ART as determined by rates of CD4 cell count increase and risk of immunological non-response.

## Methods

### Study population

We studied patients accessing ART at the Gugulethu Community Health Centre, Cape Town, South Africa [5]. The vast majority of patients receiving treatment at this clinic live in conditions of low socioeconomic status and HIV transmission is predominantly heterosexual. The South African national ART programme guidelines are based on the World Health Organisation (WHO) 2002 recommendations [17], with criteria for ART including those with a prior AIDS diagnosis (WHO stage 4 disease) or a blood CD4 cell count <200 cells/μl.

The first-line ART regimen was comprised of stavudine, lamivudine plus a non-nucleoside reverse transcriptase inhibitor (efavirenz or nevirapine). The second-line regimen for those failing first-line treatment was comprised of lopinavir/ritonavir, zidovudine and didanosine. Treatment adherence exceeds 90% at one year [18]. All treatment was free of charge and there were no interruptions in drug supply. All patients with CD4 counts <200 cells/μl received daily cotrimoxazole prophylaxis; dapsone was used as an alternative.

### Data collection

Plasma HIV-1 load was measured at baseline and 4 monthly during treatment using Versant™ HIV-1 RNA 3.0 branched chain DNA assay (Bayer HealthCare, Leverkusen, Germany) with a lower limit of detection of 50 RNA copies/ml. Blood CD4 cell counts were measured at the same time-points by flow cytometry using FACSCount™ (Becton Dickinson Inc., Franklin Lakes, NJ, USA). These assays were all performed in a single nationally accredited laboratory which has rigorous quality assurance procedures.

Structured clinical and laboratory records were maintained on all patients screened on entry to the ART programme and this information was transferred on a weekly basis to a computer database. Data were analysed from the start of the programme in September 2002 until data censorship in August 2005. All treatment-naive patients aged over 15 years and with at least a 16-week follow-up time point were included in the analysis. Study of these patients was in compliance with the Declaration of Helsinki and was approved by the Research Ethics Committee of the University of Cape Town; all patients enrolled gave written informed consent.

### Data analysis

Data were analysed using Stata version 9.0 (College Station, Texas, USA). We calculated absolute responses in CD4 cell counts during three intervals (from baseline to 16 weeks of ART, 16 to 32 weeks, and 32 to 48 weeks), as well as rates of CD4 cell increase (cells/μl/month) during each interval. The first interval was used as an estimate of the initial (phase 1) response to ART. The CD4 cell count responses observed during the latter two intervals (16 to 32 and 32 to 48 weeks after treatment initiation) were very similar, and were combined into a single measure of the second phase of CD4 cell responses (phase 2).

We further evaluated CD4 cell responses in the following ways: i) whether patients failed to attain an absolute CD4 cell count increase from baseline of at least 50 cells/μl at 48 weeks (defined as immunological non-response); ii) whether patients achieved an absolute CD4 count of 200 cells/μl at the 48 week visit; iii) and whether patients had achieved an absolute CD4 cell count of 500 cells/μl at 48 weeks (super-responders).

In bivariate analyses, medians were compared using Wilcoxon rank-sum and sign-rank tests; proportions were compared using chi-square tests. All statistical tests were two-sided at alpha = 0.05. Separate multiple linear regression models were used to examine factors associated with rates of CD4 cell count increase per month during the first and second phases. Baseline CD4 cell counts were categorised as follows: <50, 50–99, 100–149 and >150 cells/μl. Multiple logistic regression was used to assess factors associated with the binary outcomes of a CD4 cell increase of ≥ 50 cells/μl and ≥ 100 cells/μl during the 48 weeks of ART, as well as achieving an absolute CD4 cell count of ≥ 200 cells/μl during follow-up. Variables were included in multivariate models if they demonstrated a persistent association with the outcome of interest, or if their removal affected appreciably associations involving other variables [19].

## Results

### Baseline characteristics and follow-up

Of 698 individuals who commenced ART between September 2002 and April 2005, 596 (85%) had completed a 16-week follow-up visit at the time of data censorship, 34 (5%) were awaiting this appointment, 48 (7%) had died and 20 (3%) were either transferred out or were lost to follow-up. Of the 596 individuals who met the inclusion criteria for the study, 580 (97%) remained within the programme at study censorship, 11 (2%) died and 7 (1%) were lost from the programme.

At baseline the median age was 32 years (IQR, 28–38) and 75% were female. The median plasma viral load was 4.88 log_10 _RNA copies/ml (IQR, 4.50–5.27) and 58% of patients had a viral load >100,000 copies/ml. The median blood CD4 cell count was 97 cells/μl (IQR, 50–153) and the proportions of patients with CD4 cell counts within the ranges <50, 50–99, 100–149 and ≥ 150 cells/μl were 25%, 26%, 23% and 26%, respectively. Eighty per cent of patients had symptomatic disease, with 53% and 27% of patients having WHO stages 3 and 4 disease, respectively. During follow-up, only 7 (1%) patients switched to the second-line drug regimen.

### Virological and CD4 cell responses to ART

**Figure 1:**
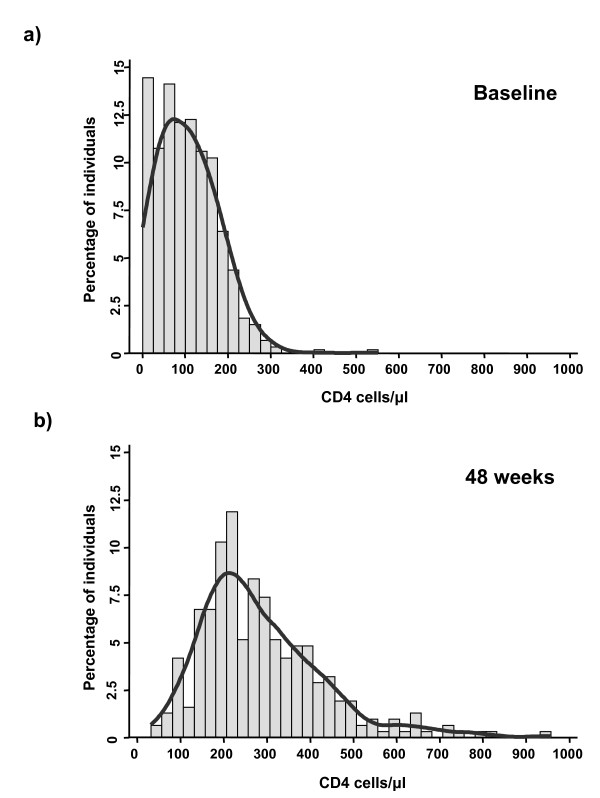
Graphs showing the frequency distribution of absolute blood CD4 cell counts (a) at baseline (n = 596) and (b) after 48 weeks (n = 311) of antiretroviral treatment. Smoothed curves have been fitted.

Virological responses to treatment were excellent with viral load suppressed <400 copies/ml in ≥ 94% of patients at each of the follow-up time-points (Table 1). As a result, the frequency distribution of blood CD4 cell counts within the cohort shifted markedly from baseline over 48 weeks of treatment (Fig. 1). Whereas the proportion of individuals with CD4 cell counts <100 cells/μl was 51% at baseline, this proportion decreased to only 4% by week 48 of treatment. The median CD4 cell count increased almost 3-fold from baseline, reaching 261 cells/μl after 48 weeks ART (Table 1).

**Table 1:** Changes in blood CD4 cell counts and plasma viral load during ART.

	**Baseline**	**Week 16**	**Week 32**	**Week 48**
**No. of patients**	596	596	404	311
**Virological Response**				
Median VL	75,858	<50	<50	<50
Number (%) patients wilh VL ≥ 400	585 (98)	31 (5)	26 (6)	18(6)
Number (%) patients with VL ≥ 50	591 (99)	117 (20)	74 (18)	54 (17)
**CD4 Cell Count Response**				
Median (IQR) CD4 cell count (cells/μl)	97 (50–153)	199 (140,314)	226 (162, 314)	261 (193, 363)
Median (IQR) CD4 cell slope in preceding interval (cells/μl/month)	-	25.5 (12.7, 42.9)	7.5 (-4.6, 19.8)	7.9 (-3.0, 20.0)

VL = viral load (copies/ml)

The rate of CD4 cell count increase in the first 16 week period greatly exceeded the rates in both the 16–32 week and 32–48 week intervals, but the rates did not differ comparing the latter two intervals. Thus, the pattern of CD4 cell count increase was divided into 2 phases: a ra–pid phase 1 (0–16 weeks; median = 25.5 cells/μl/month) and a slower phase 2 (16–48 weeks; median = 7.7 cells/μl/month).

Baseline characteristics and rates of CD4 cell count change in phase 1 and phase 2 did not differ when comparing the results of analyses of all eligible patients with those restricted to subjects who had data for every time-point (n = 292); this was also the case for all subsequent stratified analyses. Use of data from the larger cohort (n = 596) was therefore validated.

### Effect of baseline CD4 cell count on rates of CD4 cell increases

**Figure 2:**
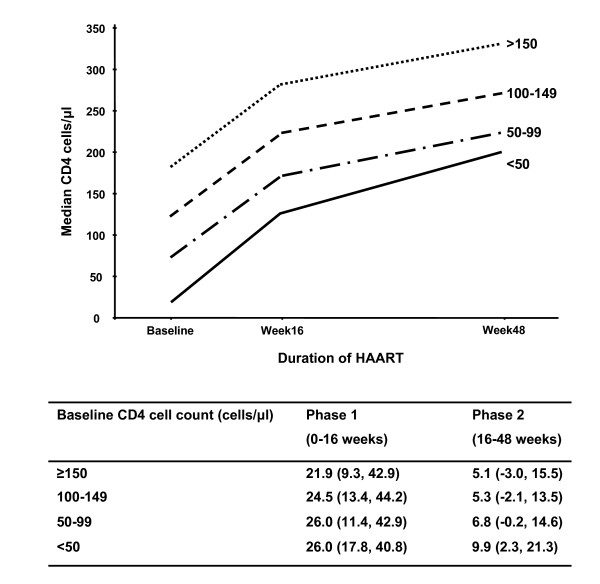
Graph showing the median CD4 cell counts at baseline and during ART stratified by baseline CD4 cell count. Below the graph, the median (IQR) rates of CD4 cell increase (cells/μl/month) are given for phase 1 (0 – 16 weeks) and phase 2 (16–48 weeks) of immune recovery.

Rates of phase 1 CD4 cell increase were similar across all CD4 cell count strata (Figure 2). Subsequent multivariate analysis found that baseline plasma viral load was the single factor independently associated with phase 1 CD4 cell recovery (Table 2). This association was strong; among those with a baseline viral load <5 log_10 _copies/ml the phase 1 CD4 cell slope was 21.3 cells/μl/month compared to 31.0 cells/μl/month among those with baseline viral load of >5 log_10 _copies/ml (P < 0.001).

**Table 2:** Multiple linear regression models estimating the average change in CD4 cell count per month (CD4 slope). Separate models analyse the first (0 – 16 weeks) and second (16 – 48 weeks) phases of immune recovery during ART.

		**Phase 1 (0–16 weeks) CD4 slope (n = 596)**			**Phase 2 (16–48 weeks) CD4 slope (n = 311)^1^**		
		**Mean**	**(95% CI)**	**P value**	**Mean**	**(95% CI)**	**P value**
**Age (years)**	<30	1.0			1.0		
	30–39	-2,27	(-6.71,2.17)	**0.32**	-2.18	(-5.98, 1.63)	**0.26**
	>40	-2.17	(-7.87, 3.54)	**0.46**	-7.31	(-12.11, -2. 50)	**0.003**
**Sex**	Female	1.0			1.0		
	Male	-3.60	(-8.22, 1.02)	**0.13**	-0.94	(-4.98, 3.10)	**0.65**
**Baseline CD4 coimt (cells/μl)**	>150	1.0			1.0		
	100–149	3.85	(-1.63, 9.33)	**0,17**	-2.17	(-7.09, 2.76)	**0.39**
	50–99	3.53	(-1.84, 8.91)	**0.20**	0.93	(-4.22, 6.08)	**0.72**
	<50	2.34	(-3.31, 7.98)	**0.42**	8.55	(2.39, 14.71)	**0.007**
**WHO clinical stage**	1 & 2	1.0			1.0		
	3	1.79	(-3.33, 6.91)	**0.49**	-0.70	(-5.40, 4.00)	**0.77**
	4	0.55	(-5.39, 6.49)	**0.86**	-1.76	(-6.93, 3.41)	**0.50**
**Baseline viral load (copies/ml)**	<5 log_10_	1.0			1.0		
	>5 log_10_	11.14	(7.10, 15.18)	**<0.001**	2.21	(-1.37, 5.80)	**0.23**

^1 ^The Phase 2 model included as covariates all variables shown, as well the phase 1 CD4 cell slope, the CD4 cell count at 16 weeks and the viral load at 16 weeks (see text).

**Figure 3:**
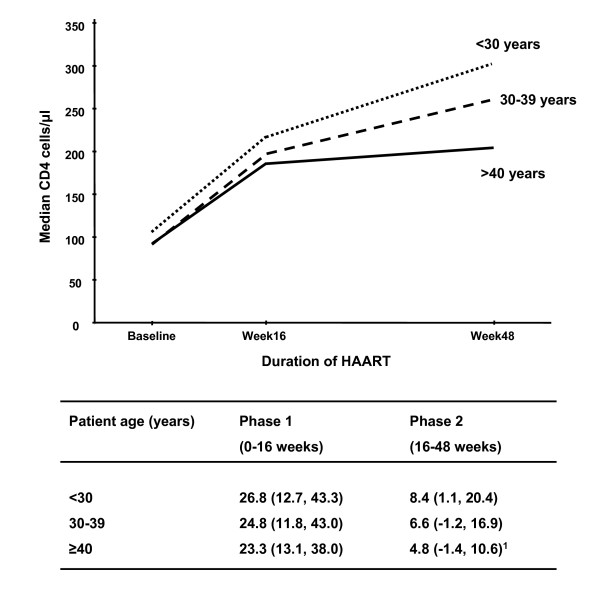
Graph showing the median CD4 cell counts at baseline and during ART stratified by age. Below the graph, the median (IQR) rates of CD4 cell increase (cells/μl/month) are given for phase 1 (0 – 16 weeks) and phase 2 (16–48 weeks) of immune recovery.

Contrary to our initial hypothesis, the rate of phase 2 CD4 cell increase was greater among those with a baseline CD4 cell count <50 cells/μl compared to those with higher baseline counts (Figure 2). This association was highly significant in multivariate analysis (Table 2). The rate of phase 2 CD4 cell increase was also greater among younger compared to older patients (Figure 3). Multivariate analysis confirmed that both low baseline CD4 cell count and younger age were the only baseline characteristics that were significantly associated with higher rates of phase 2 CD4 cell recovery (Table 2).

In the multivariate model predicting rates of phase 2 CD4 cell increase, factors associated with the response to ART during the first 16 weeks were also included. A lower rate of phase 1 CD4 cell increase and full suppression of viral load at 16 weeks were both strongly associated with greater rate of phase 2 CD4 cell increase. Viral load suppression <400 copies/ml at 16 weeks was associated with a subsequent rate of CD4 cell increase of 6.8 cells/μl/month compared to 0.7 cells/μl/month among those whose viral load remained >400 copies/ml (P < 0.001).

### Baseline CD4 cell count and risk of immunological non-response

We next examined whether baseline CD4 cell count was a risk factor associated with immunological non-response (defined as a CD4 cell increase of <50 cells/μl at 48 weeks). Among those followed to 48 weeks (n = 311), the blood CD4 cell count increased by <50 cells/μl among 30 (9.7%) patients; of these, the viral load was suppressed <50 copies/ml among 22 patients, representing a treatment discordance rate of 7%. Contrary to our initial hypothesis, low baseline CD4 cell counts were not associated with increased risk of immunological non-response. Among patients with baseline CD4 cell counts of <50, 50–99, 100–149 and 150 cells/μl, the proportions of patients who were immunological non-responders were 5%, 4%, 11% and 19%, respectively. Furthermore, in multivariate analyses, an increment of <50 cells/μl was independently associated with higher baseline CD4 cell count as well as older age, lower baseline viral load, and a viral load >400 copies/ml at any follow-up time-point (Table 3).

**Table 3:** Results of logistic regression models predicting overall change in blood CD4 cell count during ART. Responses are defined as either (i) the risk of immunological non-response (an increase of <50 cells/μl) or (ii) failure to attain an absolute CD4 cell count of ≥ 200 cells/μl after 48 weeks ART.

		**Risk of immumological non-respouse at 48 weeks**			**Risk of failure to attain CD4 cell count of ≥ 200 cells/μl at 48 weeks**		
		**OR**	**(95% CI)**	**P value**	**OR**	**(95% CI)**	**P value**
**Age (years)**	<30			1.0	1.0		
	30–39	3.15	(1.09, 9.15)	**0.035**	1.34	(0.66, 2.72)	**0.41**
	>40	3.88	(1.13, 13.39)	**0.032**	3.52	(1.55, 7.99)	**0.003**
**Sex**	Female	1.0					
	Male	0.42	(0.13, 1.39)	**0.16**	1.73	(0.90, 3.35)	**0.10**
**Baseline CD4 count**	>150	1.0			1.0		
	100–149	0.63	(0.23, 1.72)	**0.37**	2.49	(0.89, 6.96)	**0.08**
	50–99	0.18	(0.05 0.70)	**0.013**	5.97	(2.29, 15.58)	**<0.001**
	<50	0.18	(0.05, 0.72)	**0.016**	12.11	(4.43, 33.14)	**<0.001**
**WHO clinical stage**	1 & 2	1.0			1.0		
	3	1.12	(0.37, 3.40)	**0.84**	1.32	(0.54, 3.26)	**0.54**
	4	2.11	(0.63, 7.03)	**0.22**	1.19	(0.45, 3.17)	**0.72**
**Baseline viral load**	<5 log_10_	1.0			1.0		
	>5 log_10_	0.21	(0.07, 0.66)	**0.008**	0.47	(0.26, 0.86)	**0.014**
**Follow-up viral load**	<400 all visits	1.0			1.0		
	>400 any visit	4.25	(1.30, 13.87)	**0.017**	3.00	(1.14, 7.90)	**0.026**

### Baseline CD4 cell count and failure to attain a CD4 cell count of ≥ 200 cells/μl

We next determined factors associated with failure to attain an absolute CD4 cell count of 200 cells/μl at 48 weeks. Although patients with a baseline CD4 cell count <50 cells/μl had similar rates of phase 1 CD4 cell recovery and greater rates of phase 2 CD4 cell recovery compared to those with higher baseline counts, such patients nevertheless had a reduced likelihood of attaining a CD4 cell count of 200 cells/μl at 48 weeks (Table 3). The proportions of patients with baseline CD4 cell counts of <50, 50–99, 100–149 and 150 cells/μl who failed to attain a CD4 cell count of 200 cells/μl at 48 weeks were 49%, 35%, 18%, and 9%, respectively. Failure to attain 200 cells/μl was also significantly associated with older age, lower baseline plasma viral load, and a viral load >400 copies/ml at any follow-up time-point (Table 3).

### Super-responders

Of 311 patients studied out to 48 weeks, 21 (6.8%) achieved an absolute CD4 cell count of >500 cells/μl. These super-responders were principally characterised by age <40 years and by all having follow-up viral loads persistently suppressed <50 copies/ml and a CD4 cell count of 150 cells/μl after 16 weeks of ART. This group of patients had a wide distribution of baseline CD4 cell counts, and included among them were 4 (19%) who had baseline CD4 cell counts of <50 cells/μl. Thus, a low baseline CD4 cell count did not preclude patients from having an excellent immunological response.

## Discussion

To our knowledge this is the first analysis to examine the determinants of CD4 cell count recovery among patients receiving ART in resource limited settings. These data indicate that those with baseline CD4 cell counts <50 cells/μl had similar rates of phase 1 CD4 recovery (0–16 weeks) and greater rates of phase 2 recovery (16–48 weeks) compared to rates among those with higher baseline CD4 cell counts. Moreover, those with the lowest baseline counts were least likely to be immunological non-responders to ART. Despite these observations, those with baseline CD4 cell counts <50 cells/μl were nevertheless least likely to attain a CD4 cell count 200 cells/μl at 48 weeks. Taken together, these results suggest that although patients with very low baseline CD4 cell counts retain capacity for similar or slightly greater rates of CD4 cell count recovery compared to those with higher counts, they are nevertheless likely to require a longer period of time to attain a CD4 cell count threshold of 200 cells/μl. Thus, a prolonged period below a 'safe' CD4 cell count threshold rather than a diminished rate of immunological recovery is likely to underlie the high rates of morbidity and mortality observed among those with advanced disease during the early months of ART [4,5,8].

The findings of this analysis are strengthened by the relatively homogeneous study population receiving treatment at a single facility using standardised clinical protocols. Patients were all ART-naïve and received a standard triple-drug regimen with uniform follow-up time points. Quality-assured laboratory assays were all performed in a single nationally accredited laboratory. In contrast, previous studies of the determinants of CD4 cell count recovery have examined heterogeneous study populations in multiple centres and used diverse treatment regimens. Moreover, these studies included many patients with prior ART exposure [20-22] and some included only those who maintained suppression of plasma HIV load [9,23,24]. Our patient population was treated under the government ART roll-out programme and data are therefore likely to be generalisable to other ART programmes in sub-Saharan Africa. Our study is limited to analysis of CD4 cell recovery during the first year of ART and long-term outcomes and their sustainability remain to be determined. Moreover, in the present analysis we have examined recovery of CD4 cell counts but not CD4 cell functional responses.

The immunological response to ART among those with low CD4 cell counts was excellent with the proportion of patients with a CD4 cell count <100 cells/μl decreasing from 51% at baseline to just 4% at 48 weeks. However, our most important finding was that in multivariate analysis baseline CD4 cell counts <50 cells/μl were independently associated with similar rates of phase 1 CD4 cell recovery and greater rates of phase 2 CD4 cell recovery compared to individuals with higher baseline CD4 cell counts. This has not been clearly highlighted in previous publications from Europe and North America although comparison of our data with previous studies is difficult in view of differing cohort compositions. However, this overall observation is consistent with the findings of Bennett *et al.*[21], and Le Moing *et al. *showed a similar but non-significant trend when comparing patients with baseline CD4 cell counts <200 cells/μl with those with higher counts [20]. Kaufmann *et al. *found that CD4 cell increases in the first year of ART were similar comparing those with baseline counts <100 cells/μl with those with counts 100–199 cells/μl [22].

Survival bias could potentially have affected our findings. We have previously shown in this cohort that patients with the lowest baseline CD4 cell counts had a higher risk of death [5] and immunological non-responders or poor responders may have a greater mortality risk, leading to a survivor effect. However, we were careful to ensure that rates of CD4 cell recovery in the CD4 cell strata did not differ when comparing analyses of the whole cohort with those for whom data points were available at every time-point. Secondly, WHO clinical stage of disease is the strongest predictor of death in this cohort [5] and yet this variable was not associated with CD4 cell responses. Thirdly, the majority of deaths occurred in the first few weeks of ART among those whose disease was simply too far advanced; such deaths probably do not reflect a lack of immunological response to ART. Finally, we have previously reported that over 20% of early deaths in this cohort are due to immune reconstitution disease [5,25]; such deaths typically occur among those with low baseline CD4 cell counts and good CD4 cell recovery. These deaths would tend to cause exactly the opposite bias. Thus, although an important consideration, we do not think that survival bias had an overall dominant effect.

We have previously shown that low baseline CD4 count at entry to an ART programme was associated with increased risks of tuberculosis and of mortality during the first year of ART [5,8]. Results from the present study suggest that these increased risks are likely to reflect an increased period of time required for such patients to achieve a 'safe' level of immune function rather than reflecting impaired rates of immune recovery and this is consistent with the findings of a study from Spain with longer term follow-up [11]. More recently we have found that risk of mortality and risk of incident tuberculosis is strongly associated with the current CD4 cell count during ART rather than the baseline count (unpublished data). Thus, if patients with profound baseline CD4 lymphocytopenia survive the initial few months of treatment and achieve full viral load suppression, then high rates of immune recovery are likely to result in a better prognosis that might have been anticipated.

Rates of phase 1 and phase 2 CD4 cell increase were similar in magnitude to those previously reported from high-income countries [9,20]. The rapid phase 1 CD4 cell recovery was strongly associated with baseline viral load as described previously [20,21]; patients with viral loads >10^5 ^log_10 _copies/ml had 11-fold greater CD4 count increases than those with lower viral loads. Immune dysregulation and immune cell activation promote sequestration of CD4^+^CD45Ro^+ ^memory T cells in lymphoid tissue; suppression of viral replication then triggers rapid redistribution of this cell pool and a reduction in apoptotic cell death during the initial weeks of ART [26,27]. A positive correlation between the plasma viral load and the degree of cell sequestration may provide a possible mechanism underlying the observation that those with the highest viral loads have the greatest initial CD4 cell increment. The fact that patients with profound CD4 lymphocytopenia have good immunological recovery during ART is likely to be important in the pathogenesis of immune reconstitution disease associated with *Mycobacterium tuberculosis*[28], *Cryptococcus neoformans*[25] that is frequently observed in this patient population.

Greater phase 2 CD4 cell recovery was strongly associated with age as reported elsewhere [9,24]. Sustained suppression of viral replication is also critical to second phase recovery [20,21] and we confirmed that viral load at 16 weeks was a strong independent predictor of subsequent immunological recovery. Ten per cent of patients were immunological non-responders (CD4 cell increment <50 cells/μl at 48 weeks) and 7% of patients had discordant responses, having immunological non-response despite a fully suppressed viral load. These rates are much lower than those reported in previous series from industrialised countries [14,29], possibly because our study only included antiretroviral-naïve patients, rates of HIV primary drug resistance in this setting are likely to be low, and because rates of treatment adherence in our cohort are very high. However, those with CD4 cell counts <50 cells/μl were least likely to be immunological non-responders. Moreover, 19% of 'super-responders' had baseline CD4 cell counts <50 cells/μl, indicating that a very low baseline CD4 cell count does not preclude an excellent CD4 cell count response to ART.

## Conclusion

Patients with the lowest CD4 counts in this setting do not have diminished capacity for immune recovery. Although patients with low baseline CD4 counts have increased risk of acute morbidity and mortality, if such patients survive the initial months of ART and fully suppress the viral load, their chances of immunological recovery are good during the first year. Future studies are required to determine the long-term prospects for immune recovery among patients treated in ART programmes in sub-Saharan Africa.

## Competing interests

The author(s) declare that they have no competing interests.

## Authors' contributions

SDL and RW initiated and designed the study. LM did the statistical analyses. LGB and RW established the study cohort and data collection systems. SDL wrote the manuscript, which RW, LM and LGB all helped to revise.

## Pre-publication history

The pre-publication history for this paper can be accessed here:


